# A Novel Approach to Characterize State-level Food Environment and Predict Obesity Rate Using Social Media Data: Correlational Study

**DOI:** 10.2196/39340

**Published:** 2022-12-13

**Authors:** Chuqin Li, Alexis Jordan, Jun Song, Yaorong Ge, Albert Park

**Affiliations:** 1 Department of Software and Information Systems, College of Computing and Informatics University of North Carolina at Charlotte Charlotte, NC United States; 2 Department of Statistics Korea University Seoul Republic of Korea

**Keywords:** obesity, social media, machine learning, lifestyle, environment, food, correlation, modeling, predict, rates, outcome, category, dishes, popular, mobile phone

## Abstract

**Background:**

Community obesity outcomes can reflect the food environment to which the community belongs. Recent studies have suggested that the local food environment can be measured by the degree of food accessibility, and survey data are normally used to calculate food accessibility. However, compared with survey data, social media data are organic, continuously updated, and cheaper to collect.

**Objective:**

The objective of our study was to use publicly available social media data to learn the relationship between food environment and obesity rates at the state level.

**Methods:**

To characterize the caloric information of the local food environment, we used food categories from Yelp and collected caloric information from MyFitnessPal for each category based on their popular dishes. We then calculated the average calories for each category and created a weighted score for each state. We also calculated 2 other dimensions from the concept of access, acceptability and affordability, to build obesity prediction models.

**Results:**

The local food environment characterized using only publicly available social media data had a statistically significant correlation with the state obesity rate. We achieved a Pearson correlation of 0.796 between the predicted obesity rate and the reported obesity rate from the Behavioral Risk Factor Surveillance System across US states and the District of Columbia. The model with 3 generated feature sets achieved the best performance.

**Conclusions:**

Our study proposed a method for characterizing state-level food environments only using continuously updated social media data. State-level food environments were accurately described using social media data, and the model also showed a disparity in the available food between states with different obesity rates. The proposed method should elastically apply to local food environments of different sizes and predict obesity rates effectively.

## Introduction

### Background

The current obesity epidemic poses critical public health challenges. Obesity is a major risk factor for other chronic diseases, such as cardiovascular disease, cancer, diabetes, and respiratory disorders, which account for 60% of the deaths worldwide [1]. Excessive body weight has resulted in a medical expenditure of US $100 billion per year [2,3]. From 2017 to 2018, the prevalence of obesity among adults in the United States was 42.4% [4]. This number has more than tripled since the 1960s. From 1960 to 1962, the obesity rate was 13.4% [5].

Environmental factors, including the types of available food, have been identified as one of the main drivers of obesity [3,6,7]. It was reported that American adults have developed a preference for dining out with friends as opposed to cooking at home [8]. This preference could potentially impact health outcomes. A market research survey conducted in 2017 found that those who frequent fast-food restaurants are more concerned about the value of money spent and service speed than the actual healthiness of the food offered [8]. This indication that the perceived food availability tends to affect dietary outcomes has been furthered only in a literature review conducted by Caspi et al [9]. Those who live in areas highly saturated with high-fat food items tend to have health issues. In addition, those who live in lower-income areas are more likely to have at least one diet-related health issue [9]. In the United States, people tend to eat what is affordable and available to them. Environments littered with low-cost, high-fat foods tend to be obesogenic. With food expenditures for dining out increasing in recent years [3,10], understanding the food environmental factors is critical in counteracting the obesity epidemic and understanding related human behavior.

Recent studies have suggested that the local food environment can be measured by the degree of food accessibility [6,11]. These studies measured food accessibility using survey data [12], yellow pages phone books [13,14], and local business directories [15]. A limited number of samples and a significant delay between the collection and reporting of data are major limitations of these traditional methods [9]. With the proliferation of social media, the data from social media are organic, continuously updated, and generally free for large-scale collection. Several studies have used social media data to learn food environments by estimating the calorie density of the foods mentioned in tweets [16] or using the linguistic variables from tweets [17-19] to predict the local obesity rate.

In this study, we leveraged large-scale social media data sets to measure food environments at the state level and predict state-level obesity rates. It remained unclear whether we could characterize state-level food environments from the perspective of *concept of access* and predict obesity rate according to the perspective using publicly available social media data. Obesity rate was obtained from the Behavioral Risk Factor Surveillance System (BRFSS), the nation’s premier system for collecting data to improve public health.

The primary aim of this descriptive study was to understand the impact of food environment on obesity with three specific research questions (RQs):

1. RQ1: Is there a difference between the available food categories in low and high obesity prevalent states?
2. RQ2: How can we use calorie information to quantify state-level food environments?
3. RQ3: Can we predict state-level obesity rate using publicly available social media data?

We reported our novel approaches and findings. To date, to our knowledge, our study is the first to combine information from Yelp and MyFitnessPal (MFP) to learn about the local food environment and then to predict the state-level obesity rate.

### Related Work

#### Calorie With Obesity

An increase in daily calorie consumption is a major cause of the obesity epidemic [7]. The daily calorie intake rose by >500 calories in adults and >150 calories in children between 1977 and 2006 [20,21], as did the portion size in restaurants [22]. Exposure to a larger portion size increases the risk of increasing calorie intake and, therefore, weight gain [23]. Similarly, calorie intake is also affected by a higher number of local dining options. For example, the prevalence of obesity is lower in areas with supermarkets and higher in areas with higher numbers of fast-food restaurants [12].

Analysis of the data on environmental changes has identified the changes on food environment as a potential cause for the increase in caloric intake. The enormous growth in dining out, particularly at “fast-food” outlets, is a trend that has received a lot of attention. Fast-food outlets increased from approximately 30,000 in 1970 to >233,000 locations in 2004 in the United States [3]. Fast food can contribute to increasing obesity rate because it generally provides food that is poor in micronutrients, low in fiber, high in glycemic load, and excessive in portion size and calorie [24,25].

#### How to Characterize or Quantify Local Food Environment

Food access dimensions can be conceptualized using the *concept of access* proposed by Penchansky and Thomas [26]. The concept of access uses 5 dimensions to conceptualize the local food environment, namely availability, accessibility, affordability, acceptability, and accommodation [9,26]. Availability refers to the relationship between the number and type of food suppliers available to customers. Accessibility refers to the relationship between the location of food suppliers and the location of customers, which is more geographically inherent than availability. Accessibility could be measured by the travel time and distance between food suppliers and customers. Affordability refers to the price customers need to pay for the food. Acceptability refers to customers’ attitudes toward a business. Accommodation is another dimension of access, which assesses whether local businesses accept and adapt to local customers’ needs.

A variety of approaches have been used to learn about local food environments by measuring the degree of food access. These approaches typically fall into 2 categories. The first category consists of methods that capture food environment by relying on respondent-based data. The accessibility of food stores was asked about in surveys or questionnaires. The methods in the second category used the geographic information system (GIS) technology. GIS measures the buffer distance to food stores or the density of food stores in an area [12-15]. By 2007, the GIS-based measures of food environment outnumbered the respondent-based measures, and the trend of using GIS measures continued [9,27,28]. The GIS data used in previous studies primarily used publicly available data sets, such as the United States yellow pages phone book [13,14], published data from the local Departments of Environmental Health and state Departments of Agriculture [12], and local business directories [15]. A major limitation of these traditional data collections is that they are cost-ineffective and labor intensive; moreover, these methods can only gather a limited number of samples, and there is a significant delay between the collection and reporting of data [9]. In the following section, we will illustrate quantifying the environment using social media data.

#### Using Social Media Data to Learn Obesity-Related Factors or Predict the Obesity Rate

Social media is used to characterize social factors [29] and food environment in relation to obesity. Nguyen et al [16] characterized food environment by calculating the calorie density of the foods mentioned in tweets and the percentage of each food theme out of all food-related Yelp entries from that state. They found that Twitter and Yelp posts that were indicative of higher caloric foods were related to higher mortality, higher prevalence of chronic conditions, and worse self-rated health [16]. Researchers also tried to understand healthy and unhealthy food images shared on social media in relation to obesity [30]. They created an image classifier and tested it out to classify Twitter images into definitively healthy, healthy, unhealthy, and definitively unhealthy categories. Social media was also used to understand obesity-preventive factors, such as physical activity [31]. The authors described how individuals organically use social media to encourage and sustain physical activity for obesity prevention.

Social media can also be used to predict obesity rate. Fried et al [17] presented “the predictive power behind the language of food on social media.” They collected the food-related tweets that contained meal-related hashtags: dinner, breakfast, lunch, brunch, snack, meal, and supper. Then, they used the lexical feature from the bag-of-words model and topic features obtained from latent Dirichlet allocation to predict whether a state’s obesity rate is above or below the national median. Their best model reached an accuracy of 80.39% in predicting overweight. Culotta [18] used the linguistic variables (Linguistic Inquiry and Word Count and PERMA) from tweets and demographic variables to predict health-related statistics for the 100 most populous counties in the United States. The Pearson correlation for obesity between the predicted and real rates was 0.64. Abbar et al [19] conducted a study similar to the one by Culotta [18]. Abbar et al [19] used the linguistic variables (Linguistic Inquiry and Word Count), food features, average calorie per serving for food, and demographic variables from food-related tweets to predict county-wide obesity rate, achieving a correlation of 0.775 for obesity. Public posts about food and eating behaviors may spread through social networks [32]. These studies demonstrated a successful application of Twitter data in predicting state health outcomes. Although Yelp data together with Twitter data have been used to characterize food environment by Nguyen et al [16], no previous study has been found to use Yelp and MFP data to predict state obesity.

## Methods

### Data Collection

Our study used 3 data sources: (1) Yelp, (2) MFP, and (3) BRFSS. The data used in this study to describe state-level food environments were collected by the research team via the Yelp application programming interface (API) [33] and the web scraping tool, BeautifulSoup.

Yelp is a leading crowd-sourced review site in the United States that allows users to search for restaurants and local businesses [34]. Users can post reviews and upload photos concerning a business’s foods and services, which makes Yelp a location-based social media platform. To date, Yelp [35] ranks 52nd in the United States and 231st worldwide based on internet traffic and engagement [36].

The Yelp API allows users to search and query Yelp for more than 50 million businesses in 32 countries [33]. To obtain the data for this study, we converted 5-digit US zip codes to latitude and longitude coordinates and then queried the detailed business content via the Yelp API by searching the businesses near the provided locations. The data were collected in September 2020 and consisted of the profiles of 353,431 businesses in the United States.

**Figure 1:**
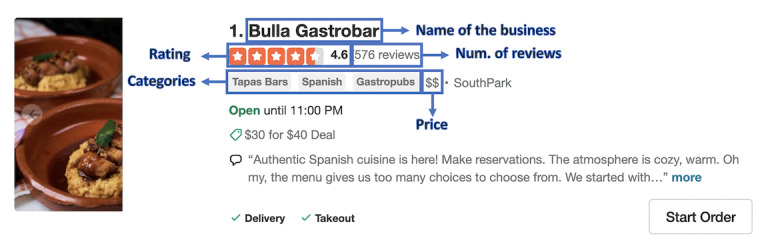
Example of the Yelp business list page.

An example of a restaurant’s listing on Yelp [35] is shown in Figure 1. As shown in Figure 1, the profile of each business includes its name, average rating, price level, and categories and the number of reviews it has received. Each business can choose up to 3 terms (categories) to describe its services and offerings. The queried business profile returned by the Yelp API not only contains the mentioned fields but also includes other details of the business, such as the business ID, address, URL to the business’s home page on Yelp [35], photos, and hours of operation. It is worth noting that chain businesses can have the same name, but each location has its unique business ID.

Yelp publishes reviews of many service businesses, such as restaurants, hospitals, and recreational activities. We removed businesses that were not related to the food industry in this study (eg, hardware stores). To do this, 2 independent reviewers first evaluated the relevance of each selected category to the food field independently. The 2 judgments reached 100% agreement with κ=1. A total of 226 categories were selected from 332 categories. In our collected data set, the total number of businesses is 353,431. The average rating of each business is 4.00 (SD 0.75), the average number of reviews of each business is 99.16 (SD 260.32), and the average price is US $1.60 ($ is the unit Yelp use to approximate cost per person for a meal) with an SD of 0.56.

**Figure 2:**
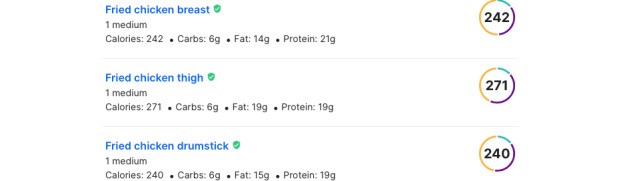
Example of the MyFitnessPal nutrition fact list page.

To understand and objectively compare these categories, we further collected data on each category’s most popular 100 restaurants nationwide and their most popular dishes for use as a proxy to estimate the caloric density of each category. We used BeautifulSoup [37] to collect popular dishes from each restaurant. We also used this web scraping tool to collect the nutritional information (ie, calories) of each popular dish from MFP. MFP is one of the most popular calorie-tracking smartphone apps worldwide with >10 million users [38]. MFP provides powerful tools to help users easily track their meals and physical activity. We collected food nutrition information by searching the food name in MFP’s nutrition database. Figure 2 shows an example search result page, which appeared when we searched the term “Fried Chicken.” We collected nutrition records for 37,295 dishes from MFP, and the total number of nutrition records is 3,110,744.

We obtained the state-level obesity rate data from the BRFSS, the nations’ state-based health surveillance system that tracks the behavioral risk factors of residents in the United States. BRFSS provided the ground truth for the prevalence of obesity via self-reported obesity data among adults in the United States by state and territory in 2019. We collected the obesity rates for 49 states and the District of Columbia, excluding New Jersey, owing to insufficient data collection by the BRFSS in 2019 [39].

### RQ1: Is There a Difference Between the Available Food Categories in States With Low and High Obesity Prevalence?

We first characterized a local food environment based on the literature and then illustrated the quantification of the environment using social media data in RQ2. We based our characterization on food access dimensions [26]. Specifically, we focused on 3 highly distinct dimensions: availability, affordability, and acceptability [9]. Availability refers to the relationship between the number and type of food suppliers available to customers. Affordability refers to the price customers need to pay for the food. Acceptability refers to customers’ attitudes toward a business.

We used the category information for each business in Yelp to calculate the availability of those food categories. Specifically, we defined the availability of a category of food as the number of available restaurants compared with the overall choices at the state level. For example, the availability of Mexican food will be equal to 1 if all the restaurants in that area sell Mexican food. Similarly, if 50% of the state’s restaurants sell Mexican food, its availability will be 50%.

After calculating the availability of all food categories, we further compared the availability of food categories between states with low prevalence of obesity and those with high prevalence of obesity. We aimed to understand the impact of local food availability, a dimension that has been widely studied [3,16,40], on the state-level obesity rate. The 2 states we selected were Colorado and Mississippi. In 2019, Mississippi had the highest obesity rate (40.8%), whereas Colorado had the lowest obesity rate (23.8%) [39]. We first calculated the availability of each category in the 2 preselected locations and further analyzed what categories of restaurants are more available in locations with high or low obesity rate. The category with the biggest availability difference was further compared by adopting dimensions from the *concept of access*.

The affordability and acceptability of the categories were then compared. Affordability refers to the food price customers need to pay. Price may affect the food choices of users. Low-income populations have a high risk of living in poor food environments and bear much of the burden of obesity and chronic diseases [14]. We estimated affordability using the price category data for each business. Here, we converted the price categories into numeric numbers for future analysis. For example, $ would have been converted to 1, and $$$$ would have been converted to 4. Acceptability refers to the client’s attitude toward the service provider. We used the average customer rating and the total number of reviews of a business to measure customers’ attitudes concerning a business. Studies have shown that consumers’ preference increases with the number of reviews [41], and consumer-generated restaurant ratings are positively associated with the web-based popularity of restaurants [42]. The businesses with higher ratings and more reviews are considered more likely to be accepted by customers than businesses with poor ratings and a limited number of reviews.

### RQ2: How Can We Use Calorie Information to Quantify State-Level Food Environments?

Because calorie intake is one of the major contributors to obesity, it is critical to understand the nutritional content of food to evaluate its effect on obesity. We evaluated the state-level food environment quantitatively using the nutritional information, specifically calorie information, collected from MFP. The categories were turned into average calories per gram for popular dishes in representative restaurants. The caloric density of each food category, which was weighted by the availability of that category in a state, became the weighted score of the caloric density of the state.

**Figure 3:**
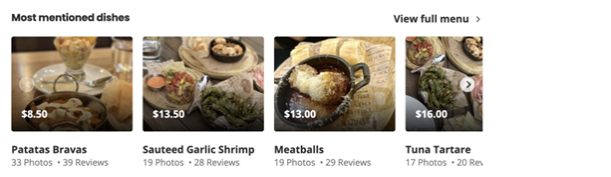
Example of the Yelp page.

To calculate the caloric density for each category, we first collected popular dishes in each category. We chose the top 100 restaurants with the highest number of reviews for each category nationwide and used the web scraping tool, BeautifulSoup, to collect the popular dishes. Yelp [35] listed the most mentioned dishes for each restaurant on the Yelp [35] pages (Figure 3). Subsequently, these popular dishes were searched in the MFP food nutrition database.

We calculated the mean calorie content of a popular dish by averaging the calories per gram of all records returned from MFP for that dish. It should be noted that the nutrition database of MFP contains a combination of foods added by MFP and foods that are added by users, and various units of measures (eg, g, gram, package, breast, oz, piece, and slices) are used. We selected gram as the unified measuring unit for comparison. We included all records that use “gram” or variations of “gram” (eg, “g,” “gr,” and “grams”) as their measuring unit.

### RQ3: Can We Predict State-Level Obesity Rates Using Calorie Information of Different Restaurant Categories and Dimensions From the Concept of Access Using Publicly Available Social Media Data?

On the basis of the results of RQ1 and RQ2, we created features from the availability, affordability, and acceptability of food categories and state weighted score for caloric density for the state-level food environment to describe the local food environment.

We classified these features into 3 sets: (1) category availability: the degree of availability of each category at the state level; (2) category affordability and acceptability: the average price of, average rating of, and average number of reviews for each category at the state level; and (3) state weighted score for caloric density: calculated weighted score for caloric density for each state. We used the scikit-learn [43] library to build our machine learning models. We applied a combination of different feature sets and used several popular machine learning models (ie, random forest regression, support vector machine regression, and XGBoost regression) for prediction. We did not use the state-of-the-art deep learning models (eg, convolutional neural network regression) in this study because we had a limited number of samples. Deep learning models would need a large sample size to outperform traditional machine learning techniques [42]. Because we were predicting obesity rate at the state level, we used the leave-one-out cross-validation. Leave-one-out cross-validation is an extreme version of k-fold cross-validation, where k is set to N. N is the number of observations in the data set. For N times, a model is created and trained on all the data except for 1 point, and a prediction is made for that point. Thus, we used information from the District of Columbia and 49 states to predict the obesity rate for the other state. Then, we repeated this 50 times while changing the predicting location. We evaluated our approach by calculating the Pearson correlation between the real and predicted obesity rates.

## Results

### RQ1: Is There a Difference Between the Available Food Categories in States With Low and High Obesity Prevalence?

We extracted business profile data of the food-related businesses located in the 2 preselected areas from the collected Yelp data. A summary of the data is presented in Table 1. First, we calculated the availability of each category in the given areas. In Mississippi, the categories with high availability included “Fast Food,” “Burgers,” “Seafood,” and “Sandwiches.” In Colorado, the categories with high availability were “Mexican,” “Breakfast and Brunch,” “Sandwiches,” and “Burgers.” The “Sandwiches” and “Burgers” categories had high availability in both Mississippi and Colorado. We further explored the differences in the availability of each category to understand the state-level food environment in both state with low obesity prevalence and state with high obesity prevalence. This was also done to highlight the importance of access to different types of food. We used the net value to measure the availability differences between the 2 different locations. The net differences were used to rank the categories in descending order.

Results for the net differences are listed in Table 2. A larger net value indicated a bigger difference. The net difference for all categories is significantly different by the *z* test. We found that 42.7% (59/138) of categories showed significant differences between the 2 states.

As shown in Table 2, a total of 40% (16/40) of categories are more significantly available in Mississippi than in Colorado (*P*≤.001), including “Fast Food,” “Buffets,” and “Donuts.” “Diners” and “Chinese” are more significantly available in Mississippi than in Colorado (*P*≤.01). “Ice Cream and Frozen Yogurt” is also found to be more available in Mississippi; however, the difference is not as significant as the aforementioned categories based on *P* values.

Alcohol-related businesses, including “Breweries,” “Cocktail Bars,” “Beer Bar,” “Wine Bars,” and “Pubs,” were found to be significantly more available in Colorado. Moreover, “Breakfast and Brunch,” “Coffee and Tea,” “Mexican,” “American (new),” “Pizza,” “Food Truck,” “Vietnamese,” “Thai,” “Asian Fusion,” “Ramen,” “Juicy Bars and Smoothies,” “Indian,” and “Cafes” were also found to be more available in Colorado than in Mississippi at *P*≤.001. “Bakeries” and “Beer, Wine, and Spirits” were more available in Colorado than in Mississippi (*P*≤.01).

“Fast Food” was found to have the biggest availability difference between Colorado and Mississippi. We further explored this category to fully understand the state-level food environment and the importance of access to different types of food. The availability of “Fast Food” in Mississippi was 13.49% (519/3845), whereas the availability of “Fast Food” in Colorado was 5.03% (358/7109). Because fast food was found to have the biggest difference in availability, we investigated the relationship between the availability of fast-food restaurants and the state-level obesity rate.

**Figure 4:**
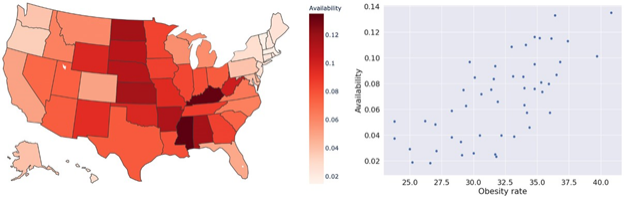
The relationship between the availability of fast-food restaurants and the state-level obesity rate. Left: availability of fast-food restaurants in a map; Right: scatter plot with the relationship between the availability of fast-food restaurants and the prevalence of state-level obesity.

We visualized the availability of fast-food restaurants in a map (Figure 4, left) and scatter plot to show the relationship between the availability of fast-food restaurants and the prevalence of state-level obesity (Figure 4, right). We found that the availability of fast-food restaurants was positively correlated with the obesity rate at the state level, with a resulting Pearson correlation of 0.676. From the heat map, we also found that the northeast had the lowest availability of fast food, and the Midwest and south had a higher availability of fast food than the west. We further adopted dimensions from *the concept of access* to compare fast-food restaurants with other restaurants.

**Figure 5:**
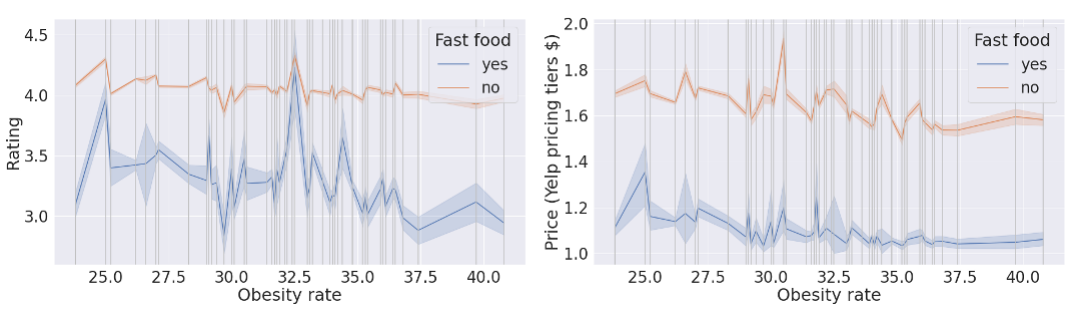
The relationship between the acceptability of restaurant type and the state-level obesity rate. Left: The relationship between the average rating of restaurant type and the state-level obesity rate; Right: The relationship between the average price of restaurant type and the state-level obesity rate.

**Figure 6:**
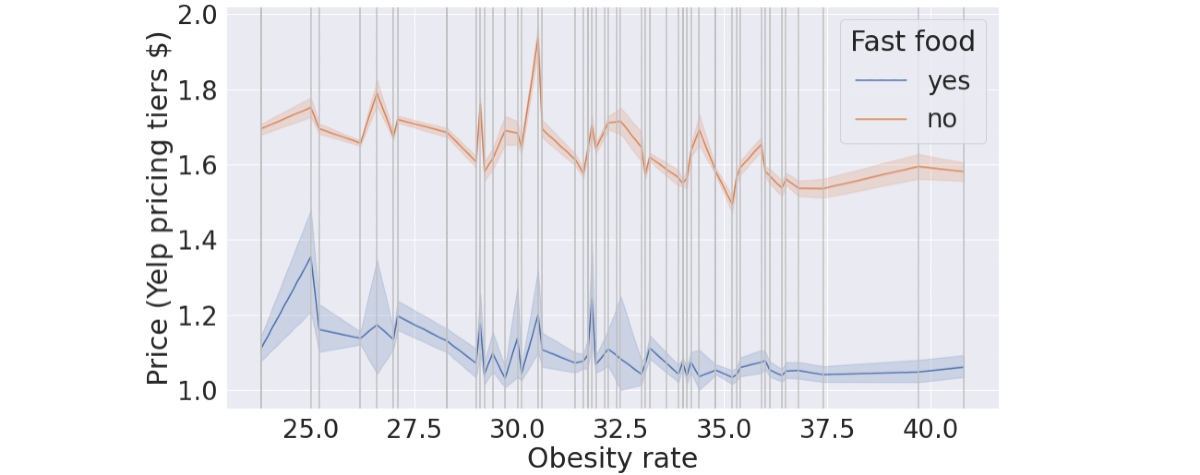
The relationship between the affordability of restaurant type and the state-level obesity rate.

We compared the acceptability (rating and number of reviews; Figure 5) and affordability (price; Figure 6) between fast-food and other restaurants.

In Figures 5 and 6, the x-axis shows the state-level obesity rate, and each vertical line represents a state with its corresponding obesity rate. The blue and orange solid lines are the average rating and average number of reviews (Figure 5) and average price (Figure 6) based on restaurant type in the state*,* and the shadow of each line is the CI. Results showed that the acceptability of fast-food restaurants was lower than that of other restaurants, irrespective of the prevalence of obesity. We found that the average rating of fast-food restaurants showed a negative relationship with the obesity rate at the state level. The residents in areas with high obesity rate gave fast-food restaurants a lower rating than the residents in areas with low obesity rate. We also found that the range of the number of reviews showed a negative relationship with obesity rate. Results on affordability showed that the price level of fast-food restaurants was lower than that of other restaurants. In addition, the prices in fast-food restaurants and other restaurants had similar trends, which indicated that the prices in fast-food restaurants are affected by the local price indices.

**Table 1:** A summary of the collected data for Colorado and Mississippi.

	Region	
	Colorado	Mississippi
Business, n (%)	7109 (2.01)	3845 (1.09)
Business categories, n (%)	215 (95.1)	142 (62.8)
Rating, mean (SD)	4.02 (0.74)	3.83 (0.96)
Reviews, mean (SD)	106.59 (197.71)	22.05 (50.14)
Price (US $), mean (SD)	1.66 (0.57)	1.50 (0.55)

**Table 2:** The 40 categories with the highest availability difference between Colorado (low obesity rate) and Mississippi (high obesity rate).

Category	Net value
Fast food^a^	0.0844^b^
Seafood^a^	0.0824^b^
Breakfast and brunch	0.0679^b^
Burgers^a^	0.0493^b^
Southern^a^	0.0470^b^
Mexican	0.0423^b^
Bars	0.0415^b^
Chicken wings^a^	0.0364^b^
American (new)	0.0353^b^
Steakhouses^a^	0.0298^b^
Pizza	0.0278^b^
Food trucks	0.0275^b^
Breweries	0.0235^b^
Buffets^a^	0.0227^b^
Coffee and tea	0.0216^b^
Cajun or creole^a^	0.0204^b^
Cafes	0.0184^b^
Cocktail bars	0.0177^b^
Convenience stores^a^	0.0175^b^
Barbeque^a^	0.0170^b^
Soul food^a^	0.0170^b^
Vietnamese	0.0156^b^
Restaurants^a^	0.0149^b^
Italian^a^	0.0115^c^
Beer bar	0.0111^b^
Thai	0.0108^b^
Bakeries	0.0105^c^
Asian fusion	0.0103^b^
Chinese^a^	0.0098^c^
Japanese^a^	0.0097^b^
Wine bars	0.0094^b^
Ramen	0.0089^b^
Pubs	0.0085^b^
Juice bars and smoothies	0.0082^b^
Tex-Mex^a^	0.0081^b^
Donuts^a^	0.0079^b^
Indian	0.0078^b^
Beer, wine, and spirits	0.0075^c^
Diners^a^	0.0075^c^
Ice cream and frozen yogurt^a^	0.0071^d^

^a^This category is more available in Mississippi, which has a higher obesity rate than Colorado.

^b^*P*≤.001.

^c^*P*≤.01.

^d^*P*≤.05.

### RQ2: How Can We Use Calorie Information to Quantify State-Level Food Environments?

The first step in quantifying a food environment was to collect the popular dishes of each category. The popular dishes of the food categories gave us an idea of why some categories were more popular in areas with high obesity. We listed the most popular dishes of the categories that we found in RQ1 to be more popular in Mississippi (Table 3) and of those that we found in RQ1 to be more popular in Colorado (Table 3). Fried food in Colorado is not as popular as in Mississippi. We collected 12,316 popular dishes for the categories that were more available in Mississippi, of which 120 (1.2%) were fried chicken. In categories that were more available in Colorado, 0.44% (114/25,910) of the popular dishes were fried chicken. The statistical test showed that the difference in proportions between the fried chicken in Mississippi and the fried chicken in Colorado was significant with *a P* value less than the significant level of .001. Similarly, the percentage of other fried foods, such as fried catfish, fried shrimp, chicken, fried steak, and fried oysters, was significantly higher in Mississippi than in Colorado. This finding is consistent with literature studies showing that the intake of fried food is associated with obesity [39].

The second step was to calculate the caloric density of each category based on the calorie information of all the available popular dishes. On average, there were 166 popular dishes per category. Table 4 shows the 5 most popular dishes per category along with the caloric density of each dish and each category. We collected up to 100 most popular (ie, highest number of reviews) restaurants in each category. A table containing the caloric densities of all categories is provided in Multimedia Appendix 1.

We further calculated the caloric density of each popular dish. The caloric density of the dishes ranged from 0.556 to 62.383, with a median value of 2.399. Bakery food had a relatively high caloric density. For example, the caloric densities of almond croissant and pecan pie were >4. Fatty meat also had a high caloric density. The caloric density of Peking duck reached 8.847, which is even higher than that of fried chicken. Cooking method also affected the caloric density. For example, the caloric density for poached egg was 1.414, for scrambled egg was 1.649, and for Eggs Benedict was 2.208; likewise, the calories per gram for fried catfish was 3.283 and for fresh fish was 1.188. Salad and soup were found with low caloric densities. The calories per gram for beet salad and French onion soup were <1 based on our calculation.

Using the calorie information of these popular dishes, we calculated the caloric density of each category by averaging the caloric density of all popular dishes. The caloric density of a category varied from 1.941 to 23.452, with a median value of

5.473. The “Cheesesteaks” was the category with the highest caloric density, followed by the “Fried Chicken” with a caloric density of 17.310. “Fruits and Veggies,” “Food Tours,” “Shaved Snow,” “Gay Bars,” and “Honey” were categories with the lowest caloric density among all food categories, with caloric density <4.

**Figure 7:**
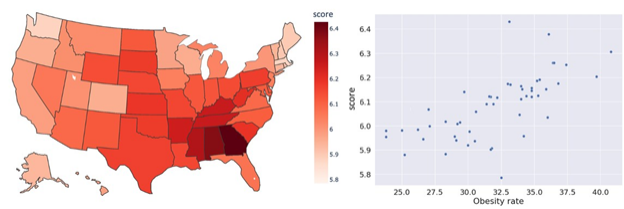
The weighted score for caloric density of each state. Left: The weighted score for caloric density in a map; Right: scatter plot with the relationship between the weighted score for caloric density and the prevalence of state-level obesity.

Finally, we converted the caloric density for each category into a weighted score for caloric density for each state. The estimated weighted score for caloric density for the states ranged from 5.786 to 6.430. Washington had the lowest estimated weighted score for caloric density, while Georgia had the highest estimated weighted score for caloric density among all the states. Colorado’s score was 5.955, and Mississippi’s score was 6.305. We performed a 2-sample *z* test between these 2 states. The result showed a significant difference with a *z* value of 12.759 and *P*<.001. The relationship between the state estimated weighted score for caloric density and state obesity rate is shown in Figure 7. The estimated weighted score for the caloric density of states calculated using our approach showed a strong positive correlation (*r*=0.671; *P*<.001) with the state-level obesity rate. A higher estimated weighted score for the caloric density of a state indicates that the state-level food environment is more prone to obesity by serving high–calorie density food. Moreover, the estimated caloric density weighted score for southern food is higher than those for other areas in the United States, especially in Georgia, Alabama, and Mississippi.

**Table 3:** The most popular dishes for categories more available in Colorado and Mississippi.

Regions	Popular dishes
Mississippi	Fried Chicken French Toast Fish Tacos Clam Chowder Crab Cakes Fried Catfish Eggs Benedict Fish and Chips Filet Mignon Beef Brisket
Colorado	French Toast Fish Tacos Pork Belly Eggs Benedict Pad Thai Fish and Chips Fried Chicken Spring Rolls Caesar Salad Avocado Toast

**Table 4:** The example of top 5 popular dishes and their caloric density for selected categories.

Category	Popular dish 1 (caloric density)	Popular dish 2 (caloric density)	Popular dish 3 (caloric density)	Popular dish 4 (caloric density)	Popular dish 5 (caloric density)	Caloric density for the category
Chicken wings	Fried chicken (2.240)	Boneless wings (1.836)	Buffalo wings (2.02)	Kimchi fried rice (3.271)	Chicken strips (2.108)	17.31
Diners	French toast (2.545)	Eggs Benedict (2.208)	Chicken fried steak (2.665)	Huevos rancheros (1.147)	Scrambled eggs (1.649)	7.289
Soul food	Fried chicken (2.240)	Fried catfish (3.283)	Sweet potato pie (2.525)	Red beans and rice (1.880)	Chicken breast (1.453)	6.337
Patisserie or cake shop	Almond croissant (4.102)	Chocolate croissant (3.926)	French toast (2.545)	Eggs Benedict (2.208)	Tiramisu (3.034)	6.298
Southern	Fried chicken (2.240)	Fried catfish (3.283)	Pecan pie (4.749)	Pork chop (1.590)	French toast (2.545)	5.667
Smokehouse	Pulled pork sandwich (2.452)	Baby back ribs (2.301)	Beef brisket (2.043)	Brisket sandwich (2.698)	Pulled pork (2.112)	5.51
American (new)	French toast (2.545)	Eggs Benedict (2.208)	Poached egg (1.414)	Fish tacos (1.498)	Beet salad (0.845)	5.047
Brasseries	French onion soup (0.808)	Pork chop (1.590)	Steak frites (2.465)	Duck confit (2.646)	Beef tartare (2.698)	4.78
Poke	Poke bowl (1.482)	Seaweed salad (3.510)	Spicy tuna (1.955)	Octopus (1.838)	Fresh fish (1.188)	4.716
Dim sum	Shrimp dumplings (1.620)	Peking duck (8.847)	BBQ^a^ pork buns (2.505)	Har gow (1.741)	Xiao Long Bao (2.419)	4.215

^a^BBQ: barbecue.

### RQ3: Can We Predict State-Level Obesity Rates Using Publicly Available Social Media Data?

We generated 3 sets of features for the prediction. The feature sets were as follows: (1) category availability, (2) category affordability and acceptability, and (3) weighted score for caloric density. Affordability and acceptability were created at the state level for the identified 226 categories. The estimated state weighted score for caloric density was calculated in RQ2. Because each state had only 1 estimated weighted score for caloric density, prediction models other than linear regression were not applicable for prediction using this set of features. For categories that did not exist in a state, we used 0 to fill in the missing values for the categories’ availability, affordability, and acceptability. Approximately 24% (11,065/46,104) of the features were filled with 0. Table 5 presents the results of comparing different prediction models with different combinations of input. We used the Pearson correlation coefficient between the actual obesity rate and predicted obesity rate to evaluate it.

The random forest model with all 3 sets of features performed the best. In addition, the Pearson correlation coefficient between the predicted and real obesity rates was 0.796, which indicates that the predicted value was correlated with the real value.

**Table 5:** Pearson correlation coefficients for different combinations of input for prediction.

Features	Linear regression	Random forest regression	SVM^a^ regression	XGBoost regression
Category availability	0.407	0.763	0.712	0.742
Category affordability and acceptability	0.402	0.776	0.593	0.743
State weighted score for caloric density	0.622	—^b^	—	—
Category availability+category affordability and acceptability	0.403	0.791	0.642	0.731
Category availability+state weighted score for caloric density	0.336	0.771	0.714	0.710
Category availability+category affordability and acceptability+state weighted score for caloric density	0.402	0.796^c^	0.643	0.708

^a^SVM: support vector machine.

^b^Not available.

^c^The best performing model.

## Discussion

### Principal Findings

In this study, we characterized food environments using the data from Yelp and MFP with innovative data collection and processing methods. We also predicted state-level obesity rates. In addition, our study contributed a new method to calculate food environment and data to estimate the calorie densities of different popular dishes and restaurant categories for future studies.

Our results showed a disparity in the available food categories between Colorado and Mississippi (ie, Colorado had a low obesity rate, and Mississippi had a high obesity rate). “Fast-food” restaurants were found to be more available in Mississippi than in Colorado. Fast-food consumption has been found to be strongly associated with weight gain and obesity [3]. Individual-level diet and weight outcomes are thought to improve in neighborhoods that have access to high-quality food [44]. Comparing the state-level food availability difference, we found that abundant access to fast-food options may contribute to a negative group-level health outcome. Although fast-food restaurants are notorious for serving high-calorie, low-nutritional foods [24,25] such as hamburgers, French fries, and fish and chips [45], some differences have been found. By comparing the popularity of fast-food restaurants with other restaurants in Figure 5, we found that fast-food restaurants always have a lower number of reviews than other restaurants. However, in the District of Columbia, the average number of reviews of fast-food restaurants is higher than that of other restaurants. This may be because more alternative fast foods are available in cities, such as salad, sushi, and poke, which are considered light and healthy [46].

In addition to using the available food category to characterize the state food environment, we also used the popular dish and nutrition content of popular dishes to quantify the state food environment. To our knowledge, this is the first study to conduct a large-scale analysis of popular dishes. We compared popular dishes in Colorado and Mississippi. We found that fried foods are more popular in Mississippi. This finding is consistent with the literature showing that the intake of fried food is associated with obesity [47]. Using the collected popular dishes, we calculated the weighted score for caloric density for each state. Similar studies exist. For example, Nguyen et al [16] quantified the state food environment by calculating the caloric density of food mentions in geo-tagged tweets. They used a list of more than 1430 popular foods and beverages from the US Department of Agriculture’s National Nutrient Database and calculated calories per 100 g for each food item [16]. Abbar et al [19] calculated the average calories by checking the calories per serving for the selected 500 food keywords. In contrast to these 2 studies, we used MFP, the biggest food database available [38], to obtain nutrition data. We collected nutrition data for 37,295 dishes, which allowed for an effective use of data points. In our study, Pearson correlation of weighted score for caloric density of states to state obesity rates was 0.671, which outperformed one of the aforementioned previous studies [19] in which the Pearson correlation of tweet caloric value to state obesity rates was 0.629.

To the best of our knowledge, our prediction model is the first to use Yelp and MFP data to predict state obesity rates. In contrast to previous studies that used Twitter data to predict obesity rate [17-19], our model using Yelp and MFP data had less selection bias. First, Twitter users are younger than the general public [48]; however, the user group of Yelp is more evenly distributed by age, with 33% of the users aged ≥55 years [49]. Second, the previous studies using Twitter data for prediction only used sampled data because of the massive amount of Twitter data. Although these studies used the same data source, their collection methods were different, which could have skewed the results.

### Public Health Implications

Our study helped us understand the impact of the food environment and related human behavior by showing the correlation between state-level food environment and obesity rate. Because of the pervasive use of smartphones and social media apps like Yelp across the country, researchers could use social media data to gain an understanding of food environments in any part of America and other countries as well. In sum, our model has the potential to evaluate food environments.

Not only does our model map out a landscape of the local food environment but it also allows us to characterize the trajectory of public health. The copious amounts of information on social media allow public health practitioners to monitor changes in food availability and population over time and use this information to predict changes in state obesity levels. Similarly, computational methods could be used to inform dieting habits at the individual level. This allows for an early intervention in areas or individuals facing the greatest risk of increasing obesity rates or becoming obese.

Our study has reiterated a few fundamental findings related to the importance of environment [9,18,19]. Our findings suggest that those who live in areas with a considerable availability of high-calorie, fast foods are more likely to be obese. This alludes to the idea that people eat what is readily available to them. Politicians and city planners could potentially use this information to develop an infrastructure of healthy food options in areas that have been traditionally concentrated with fast-food restaurants. This sort of environmental intervention could potentially influence community behavior and lead to better health outcomes.

### Limitations and Future Direction

The first limitation of our study lies in the data collection. Yelp provides substantial data for local businesses; however, the Yelp API results are restricted to 1000 results for each query. We could collect up to 1000 business data points for each zip code center up to a distance of 40 km (approximately 25 miles). In urban environments, 1 zip code may have >1000 businesses. To address this issue, we ran several rounds for each zip code and removed the duplicates. Despite this effort, missing data may skew our results, especially those about urban areas. We found a second limitation when collecting nutritional data from MFP. For each search query, MFP returned 10 pages with 10 records on each page. Some popular dishes did not have an exact match, in which case MFP returned a partially matching dish. Therefore, some caloric information may not be accurate. We averaged all the results to reduce the effects of inaccurate information. Another limitation is not capturing the actual consumption. We did not have information on the food consumed at a person’s home. In this study, we calculated the caloric density of popular dishes. Nevertheless, we found that high–caloric density food is correlated with obesity rate, consistent with a previous study that was conducted at the individual level [50]. To bolster our findings, a similar analysis should be replicated at the zip code–level to better inform the local food environment. We used the state-level food environment in this study because BRFSS provides state-level obesity rate. More granular analysis will provide a better insight into how socioeconomic status and the local food environment may be correlated with obesity [14,51-53]. The information collected and calculated in this study could also be used to fuse a personalized mobile health app to help user have a better experience with obesity prevention management. For example, a specialized dashboard [54] could be added to the mobile health app when using information from GPS to measure physical activity along with a heat map showing where a person goes within their neighborhood.

### Conclusions

This study used social media data to characterize state-level food environments. State-level food environments show a disparity in the available food between states with different obesity rates, suggesting the importance of food environment. Using the availability of different categories of food along with affordability and acceptability data captured on social media, we created a state-level obesity rate prediction model with a 0.796 correlation. Using our proposed method, public health practitioners could monitor the changes in areas that face the greatest risk of increasing obesity rates to counter the obesity pandemic.

## Appendix

Multimedia Appendix 1 The caloric density of all food categories.

## Abbreviations

API: application programming interface
BRFSS: Behavioral Risk Factor Surveillance System
GIS: geographic information system
MFP: MyFitnessPal
RQ: research question
