# Supplementary material for: A Novel Approach to Characterize State-level Food Environment and Predict Obesity Rate Using Social Media Data: Correlational Study
Source: J Med Internet Res. 2022 Dec 13;24(12):e39340. doi: 10.2196/39340 (PMC9795398; doi:10.2196/39340)
Supplement: Multimedia Appendix 1 [file jmir_v24i12e39340_app1.docx]

**Multimedia Appendix 1. The caloric density of all food categories.**

| **category** | **Caloric density for category** |
| --- | --- |
| Cheesesteaks | 23.45167446201210 |
| Chicken Wings | 17.310041614533100 |
| Wraps | 16.45398340827920 |
| Food Stands | 15.609912597949000 |
| Herbs & Spices | 11.136928756667300 |
| Cooking Schools | 10.735016492222000 |
| Bed & Breakfast | 10.495341100749500 |
| Wine Tasting Classes | 10.392633824300000 |
| Pretzels | 9.909451519166140 |
| Speakeasies | 9.40817675657083 |
| Empanadas | 9.17133716670696 |
| Wine Tasting Room | 7.81667741542251 |
| Piano Bars | 7.635336967355570 |
| Donuts | 7.476480689086060 |
| Korean | 7.37855465493137 |
| Shaved Ice | 7.371473947381680 |
| Vegan | 7.298928305353670 |
| Diners | 7.289469905968660 |
| Wineries | 7.25115139675034 |
| Candy Stores | 7.248143313846180 |
| Game Meat | 7.213190572864480 |
| Argentine | 7.212515970730290 |
| Popcorn Shops | 7.1140796704842200 |
| Barbeque | 7.061157173500050 |
| Acai Bowls | 6.82461954550189 |
| Macarons | 6.8110195646998600 |
| Middle Eastern | 6.798703191720400 |
| Pubs | 6.771389113256890 |
| Chocolatiers & Shops | 6.735912489708240 |
| Persian/Iranian | 6.712146538979440 |
| Basque | 6.609311526558660 |
| Pakistani | 6.609131856446620 |
| Hawaiian | 6.597510611291630 |
| Eritrean | 6.590205116210870 |
| Cafes | 6.563076207062440 |
| Internet Cafes | 6.553815378775280 |
| Burgers | 6.5355883974426900 |
| Czech | 6.528298840329150 |
| Italian | 6.516195346741980 |
| American (Traditional) | 6.470123582257670 |
| Fast Food | 6.4643336272755700 |
| Coffee Roasteries | 6.41846456228897 |
| Soul Food | 6.33709704723707 |
| Chicken Shop | 6.324942151019320 |
| Patisserie/Cake Shop | 6.297734885652010 |
| Delis | 6.275257375706340 |
| Mexican | 6.242366373481050 |
| Russian | 6.1980418685666900 |
| Personal Chefs | 6.192163376075180 |
| British | 6.18832313841777 |
| Arabian | 6.181144205508910 |
| Beer, Wine & Spirits | 6.172750846125510 |
| Pasta Shops | 6.1592449846344400 |
| German | 6.131208104541560 |
| Beer Bar | 6.120439481138630 |
| Polynesian | 6.110823699080330 |
| Tex-Mex | 6.079456955964780 |
| Butcher | 6.057187101377830 |
| Hungarian | 6.032949796136730 |
| Wine Bars | 6.024275703503040 |
| Convenience Stores | 6.017254816092240 |
| Japanese | 6.014113991083370 |
| Coffee & Tea | 5.967601085610970 |
| Mediterranean | 5.953900834442530 |
| Desserts | 5.9312173971410100 |
| Polish | 5.891291263604190 |
| Street Vendors | 5.873466463816160 |
| Bakeries | 5.871736268042050 |
| Hot Dogs | 5.8394784498199900 |
| Izakaya | 5.838129859173210 |
| Supper Clubs | 5.830291223390950 |
| Guamanian | 5.813559835327910 |
| Latin American | 5.812650402971170 |
| Armenian | 5.8082273823500300 |
| Ice Cream & Frozen Yogurt | 5.803409182505700 |
| Dinner Theater | 5.801810868481200 |
| Waffles | 5.786461607262140 |
| Brazilian | 5.77840355676942 |
| Gastropubs | 5.769347835716770 |
| Sandwiches | 5.759086115468900 |
| Tiki Bars | 5.739120175221710 |
| Pancakes | 5.72705133605195 |
| Meat Shops | 5.725768673658790 |
| Seafood | 5.717904334872660 |
| Irish Pub | 5.717052589755680 |
| Gelato | 5.692262143671430 |
| Gluten-Free | 5.670306414624310 |
| Southern | 5.6672842892913400 |
| Food Delivery Services | 5.655047510367590 |
| Specialty Food | 5.650142378390740 |
| Syrian | 5.6430226087159300 |
| Pizza | 5.633709596478550 |
| Live/Raw Food | 5.63354203938849 |
| Drive-Thru Bars | 5.626749490649330 |
| Scandinavian | 5.619364809907800 |
| Dominican | 5.616916546942670 |
| Sushi Bars | 5.6035796983754400 |
| Restaurants | 5.585839114596220 |
| Cocktail Bars | 5.582985310312650 |
| Bagels | 5.577384607466340 |
| Food Court | 5.5752092898709200 |
| Health Markets | 5.571901986838780 |
| Food Trucks | 5.522586713054540 |
| Dive Bars | 5.518394024218210 |
| Smokehouse | 5.506208139054740 |
| Modern European | 5.49109890653694 |
| Farmers Market | 5.480186514546860 |
| Do-It-Yourself Food | 5.479797115542990 |
| Lebanese | 5.473894014585540 |
| Cuban | 5.471806483289800 |
| Caterers | 5.460717975446770 |
| Grocery | 5.45492855243464 |
| Tea Rooms | 5.434575592360950 |
| Venezuelan | 5.433413338218460 |
| Distilleries | 5.4299926202902500 |
| Salad | 5.429620340523270 |
| Comfort Food | 5.4268350694900800 |
| Halal | 5.417428741069080 |
| Falafel | 5.4058804208442100 |
| New Mexican Cuisine | 5.3944004482294700 |
| Beer Gardens | 5.393223805945590 |
| Caribbean | 5.381651125806890 |
| Pop-Up Restaurants | 5.368078916456520 |
| Spanish | 5.348013650194020 |
| Sports Bars | 5.347286716152060 |
| Local Flavor | 5.3296165755619400 |
| Puerto Rican | 5.322028184630570 |
| Custom Cakes | 5.304417079337350 |
| Breakfast & Brunch | 5.281963437127100 |
| Poutineries | 5.276172988107340 |
| Vegetarian | 5.250638115315920 |
| Filipino | 5.2501694815322800 |
| Whiskey Bars | 5.231378470053330 |
| African | 5.2113436790051100 |
| Ethiopian | 5.208295135598980 |
| Vietnamese | 5.207063687708180 |
| Hot Pot | 5.203756015681750 |
| Nicaraguan | 5.194337311625660 |
| Salvadoran | 5.187948828498750 |
| Tapas Bars | 5.16585648055098 |
| Peruvian | 5.1554027315437400 |
| Breweries | 5.143478461697810 |
| Noodles | 5.140454145948200 |
| Burmese | 5.1067818394535 |
| South African | 5.102087803374150 |
| Fish & Chips | 5.101569936476790 |
| Juice Bars & Smoothies | 5.096711466407170 |
| Food | 5.069509328401080 |
| Themed Cafes | 5.0526529052761600 |
| Colombian | 5.052541767026530 |
| American (New) | 5.04709006671062 |
| French | 5.040096243542160 |
| Cafeteria | 5.036084175977840 |
| Tapas/Small Plates | 5.0251116771869300 |
| Cheese Shops | 5.0218200579548800 |
| Pan Asian | 4.977568028166950 |
| Cideries | 4.97179905611951 |
| Creperies | 4.955158271589420 |
| Cajun/Creole | 4.944505021837230 |
| Sicilian | 4.935253086082430 |
| Fondue | 4.912294919101900 |
| Greek | 4.9019058847133 |
| Soup | 4.8995896182863700 |
| Szechuan | 4.887815068151000 |
| Teppanyaki | 4.878648256806530 |
| Conveyor Belt Sushi | 4.8643326621889100 |
| Organic Stores | 4.857006957845670 |
| Portuguese | 4.855388451381640 |
| Buffets | 4.854772379379950 |
| Honduran | 4.852633689014880 |
| Beverage Store | 4.850050206715880 |
| International Grocery | 4.835055311275790 |
| Ramen | 4.821494978705710 |
| Asian Fusion | 4.811051077196040 |
| Irish | 4.804416097243310 |
| Kosher | 4.78952598702877 |
| Olive Oil | 4.786388760399240 |
| Indonesian | 4.781366466053000 |
| Brasseries | 4.7799798355754600 |
| Afghan | 4.773052267356860 |
| Bars | 4.771788112073740 |
| Kombucha | 4.7709235639008500 |
| Mongolian | 4.743645413741510 |
| Cantonese | 4.737486552887090 |
| Poke | 4.7158375888902700 |
| Laotian | 4.6986787186858900 |
| Australian | 4.68605765609611 |
| Turkish | 4.589937565239320 |
| Kebab | 4.544329669116600 |
| Bubble Tea | 4.543155654214890 |
| Japanese Curry | 4.531334568442730 |
| Seafood Markets | 4.520896977295140 |
| Taiwanese | 4.488133202884950 |
| Chinese | 4.470585951614970 |
| Tacos | 4.4535151879804600 |
| Singaporean | 4.435715355412940 |
| Imported Food | 4.429552540051580 |
| Himalayan/Nepalese | 4.4037620269744100 |
| Cooking Classes | 4.3674246651326200 |
| Indian | 4.360702675295330 |
| Public Markets | 4.332507432542550 |
| Cupcakes | 4.2981569146495300 |
| Hong Kong Style Cafe | 4.2805672785392000 |
| Moroccan | 4.268937992347190 |
| Cambodian | 4.246413552448040 |
| Malaysian | 4.244325297681280 |
| Brewpubs | 4.23099471547452 |
| Dim Sum | 4.2147004356523600 |
| Shanghainese | 4.089123985056130 |
| Thai | 4.081737509560190 |
| Fruits & Veggies | 3.93128866316493 |
| Hainan | 3.9263580920505300 |
| Austrian | 3.8864758940527800 |
| Food Tours | 3.8276116772227200 |
| Shaved Snow | 3.686936604984620 |
| Gay Bars | 2.0502207640520500 |
| Honey | 1.9411550672132600 |
